# Validating the Children’s Depression Inventory-2: Results from the Growing Up in Singapore Towards Healthy Outcomes (GUSTO) study

**DOI:** 10.1371/journal.pone.0286197

**Published:** 2023-05-25

**Authors:** Nandini Anant, Divjyot Kaur, Ranjani Nadarajan, Desiree Y. Phua, Yap Seng Chong, Peter D. Gluckman, Fabian Yap, Helen Chen, Birit Broekman, Michael J. Meaney, Yuen-Siang Ang

**Affiliations:** 1 Social and Cognitive Computing Department, Institute of High Performance Computing, Agency for Science, Technology and Research (A*STAR), Singapore, Singapore; 2 School of Health and Social Sciences, James Cook University, Singapore, Singapore; 3 Translational Neurosciences Division, Singapore Institute of Clinical Sciences, Agency for Science, Technology and Research (A*STAR), Singapore, Singapore; 4 Department of Psychiatry, Douglas Mental Health University Institute, McGill University, Montreal, Canada; 5 Yong Loo Lin School of Medicine, National University of Singapore, Singapore, Singapore; 6 Centre for Human Evolution, Adaptation and Disease, Liggins Institute, University of Auckland, Auckland, New Zealand; 7 Department of Pediatrics, KK Women’s and Children’s Hospital, Singapore, Singapore; 8 Duke-National University of Singapore Medical School, Singapore, Singapore; 9 Department of Psychological Medicine, KK Women’s and Children’s Hospital, Singapore, Singapore; 10 Department of Psychiatry, Amsterdam UMC, Location VU Medical Centre, VU University, Amsterdam, The Netherlands; 11 Brain-Body Initiative, Agency for Science, Technology and Research (A*STAR), Singapore, Singapore; Universita Cattolica del Sacro Cuore Sede di Roma, ITALY

## Abstract

Childhood-onset depression has adverse consequences that are sustained into adulthood, which increases the significance of detection in early childhood. The Children’s Depression Inventory (CDI) is used globally in evaluating depressive symptom severity in adolescents, and its second version, the CDI-2, was developed by taking into account advances in childhood depression research. Prior research has reported inconsistencies in its factor structure across populations. In addition, the CDI-2 has not yet been empirically validated with Southeast Asian populations. This study sought to empirically validate the CDI-2’s psychometric properties and evaluate its factorial structure with a Singaporean community sample of non-clinical respondents. A total sample of 730 Singaporean children aged between 8.5 and 10.5 years was used. Psychometric properties of the CDI-2, including internal consistency as well as convergent and discriminant validity, were assessed. Factor analyses were conducted to assess the developers’ original two-factor structure for a Southeast Asian population. This two-factor structure was not supported in our sample. Instead, the data provided the best fit for a hierarchical two-factor structure with factors namely, socio-emotional problems and cognitive-behavioural problems. This finding suggests that socio-cultural and demographic elements influence interpretation of depressive symptoms and therefore the emerging factor structure of the construct under scrutiny. This study highlights the need to further examine the CDI-2 and ensure that its interpretation is culture-specific. More qualitative work could also bring to light the idiosyncratic understanding of depressive symptomatology, which would then guide culture-specific validation of the CDI-2.

## Introduction

Approximately 4.4% of the world’s adult population is thought to suffer from depression [1]. The experience and effects of depression in adults have been studied in-depth and are well-documented across many different populations. For example, Ogbo and colleagues [2] found that depressive disorders in South Asian adults had a prevalence rate of 3.9%. However, a lesser-known phenomenon is childhood depression. Although several studies have shown that the effects of childhood-onset of depression can be felt through adolescence, up to and including adulthood [3], it has not been rigorously studied across various populations. Kovacs and Lopez-Duran [4], among others, reported that the persistent adverse outcomes as a result of childhood-onset of depression are more severe and acute compared to the negative consequences of late-onset depression.

The urgency to identify and treat childhood depression at an early age is thus compounded, particularly when one takes into account the fact that childhood depression can re-occur and is associated with adverse later-life outcomes such as poor academic achievements, interpersonal problems, substance abuse, and suicide [5]. Discrepancies exist in the documented prevalence of depression, especially in children. Various studies report different prevalence rates for childhood depression. For example, the prevalence of childhood depression has been reported as 10.9% among 9 to 10-year-old American children [6], 8.2% in 13 to 17-year-old American youths [7], and 17.4% in boys and 20.6% in girls among Korean adolescents aged 13–18 years [8]. These studies have not all used a standardized measure of childhood depression, which makes it harder to accurately gauge the incidence and severity of the disorder in this population. In addition, treatment for childhood depression is often not sought, as children may not recognise their symptoms as signs of depression. For example, Kaushik et al. [9] reported that on average, one in every ten children experience some form of mental distress, including depression, but less than one-third of these children are likely to report their symptoms and seek any treatment. These findings have been supported by Reavley and coworkers [10], who estimate that less than 50% of youth who suffer from depression seek treatment for it. Therefore, understanding childhood depression becomes particularly important and the onus is on adults to identify signs of depression in children.

However, it is sometimes difficult for adults to recognise depressive symptoms in children. Signs of depression in children are varied and cover the full gamut of symptoms as manifested through both internalising and externalising behaviours. This is problematic in several ways. Firstly, parents and teachers are both more likely to note externalising behaviour and misattribute them to conduct disorder or somatic issues [11], rather than depressive symptoms. Secondly, such externalising behaviour can, on some occasions, be misattributed as ’part of growing up’ and not taken seriously as a sign of childhood depression [12]. At the same time, children displaying internalising behaviours such as anxiety and withdrawal may ‘slip through the cracks’, as these symptoms are not directly observable nor as troublesome for adults as externalizing features. Thus, symptoms that are acknowledged as depression among adults may be overlooked among children [13].

Symptoms of depression can also present themselves in different ways depending on gender and developmental age [14, 15]. For example, the inability to feel pleasure in normally pleasurable activities (or anhedonia) has been reported as a common symptom of depression regardless of age. However, a child showing signs of anhedonia reports decreased interest in play, or increased boredom, while adolescents showing signs of anhedonia report decreased appetite [16]. Similarly, research has identified gender as a risk factor for depression because girls report more symptoms of depression than boys [17]. However, the gender differences in depressive symptoms in children only emerge in early adolescence, between 13 to 15 years of age [18, 19]. Given the rising concerns regarding childhood depression and the often nuanced nature of depressive symptomatology, it becomes important to consider tools specifically designed to measure depressive symptoms in children and adolescents. Researchers suggest that one way of doing so effectively is via self-report questionnaires used to evaluate the incidence and severity of depressive symptoms in children, because studies have found that children can record their symptoms, feelings and emotional states with the same degree of consistency and precision that adults record their emotional states [20].

### Children’s Depression Inventory (CDI) and CDI-2

Thus far, the most widely used self-report instrument for assessing depressive symptoms in children and adolescents is the Children’s Depression Inventory (CDI), which was developed by Maria Kovacs [21] and quickly adopted by both practitioners and researchers upon publication [22]. The CDI comprised a total score of depressive symptoms as well as five subscales into which the items of the questionnaire were divided. Despite its popularity, dozens of papers were published with factor structures of the CDI that deviated from the original five-factor structure [22]. For example, Hodges et al. [23] identified a two-factor structure of the CDI with no second-order factors among non-clinical youth, while Saylor and colleagues [24] extracted eight factors from CDI data among non-clinical children. In addition, a meta-analysis of 24 studies with data from 35 samples of youth showed very little empirical justification to support the original CDI five-factor internal structure [25]. While the number of identified factors corresponded to the original model at times, the factors were noted to be different in terms of content. Some researchers noted that the number and nature of factors extracted with the CDI vary depending on characteristics of the sample, such as the age of the respondents, the language of the questionnaire and cultural interpretations of the items [22, 26]. This is particularly relevant in light of findings that English and non-English administrations of the CDI presented conceptually different factors [25].

Several other researchers have also presented findings arguing that language and culture interact to heavily influence the CDI’s factor structure. For instance, analysing CDI data from Native American and Inuit adolescents showed a one-dimensional structure [27], while data from Nigerian adolescents who completed the CDI in English showed a two-factor structure [28] and data from Australian adolescents who also completed the CDI in English corresponded with Kovacs’s originally suggested five-factor structure [29]. These studies provide clear evidence that depression and depressive symptoms are experienced, described and conceptualised in very different ways across cultures despite the CDI being administered in a common language to all three cultures [22]. As researchers raised issues with the factor structure of the CDI, it has become evident that this measure requires further validation, especially with regard to its factor structure. In fact, upon reviewing over 300 data sets of the CDI as part of a meta-analysis, Twenge and Nolen-Hoeksema [30] suggested that a new normative sample be used, particularly in light of new findings and research in the field of childhood mental health.

Maria Kovacs reviewed both the contents of the CDI and the standardization sample and published the CDI 2^nd^ Edition–CDI-2 [31]. The CDI-2 was validated with a sample consisting of 1100 American children aged 7 to 17 years across the four major geographic regions (Northeast, Midwest, West and South) of the United States. This sample was obtained by controlling for gender and stratifying the racial distribution based on the U.S. Bureau of the Census in 2000, with Asians making up just 4.2% of the standardization sample. The clinical sample consisted of 319 children, of which Asians again comprised 4.2%. Therefore, the original factor structure identified by the CDI-2 may not be fully representative of an Asian population.

Interestingly, empirical studies continued to use the CDI to assess childhood depression, even after the CDI-2 had been published. To the best of our knowledge, only one study has thus far examined the psychometric properties of CDI-2 in an Asian context. Kim et al. [32] attempted to validate the CDI-2’s factor structure proposed by Kovacs and MHS staff [31] with a Korean non-clinical community sample. Surprisingly, while they reported a 2-factor structure in line with the original study, they identified items loading on factors that were significantly different from the original factors.

It thus remains to be seen whether the discrepancies noted in the factor structure of the CDI by various researchers are also found in the factor structure of the CDI-2. Given the inconsistencies described above and a lack of empirical studies validating the CDI-2 that confirm its factor structure across cultures, this study aimed to (a) validate the original factor structure of the CDI-2, and (b) evaluate psychometric properties of the CDI-2 with a Southeast Asian population within Singapore.

## Methods

### Participants

Data from children who participated in the Growing Up in Singapore Towards healthy Outcomes (GUSTO) birth cohort study was used for secondary analysis in this study. All the scales described below were administered to children as part of the GUSTO study when they were between 8.5 and 10.5 years old. For this study, a total of 732 children were given the Children’s Depression Inventory (2^nd^ edition). Data from two children was noted to be marked as ‘unusable’ due to indiscriminate responses, while one child’s data was incomplete, leaving a dataset from 729 participants for main analysis. Data for the Multidimensional Anxiety Scale for Children (2^nd^ edition) (*N* = 450) and Social Emotional Assets and Resilience Scales *(N* = 340), which was collected on a subset of this sample at 8.5 years of age, was also analysed.

### Measures

#### Children’s Depression Inventory, 2^nd^ edition (CDI-2) [31]

The CDI-2 comprises 28 items divided into two First-Order factors: Emotional Problems and Functional Problems. Both factors are further divided into two Second-Order factors. The Emotional Problems subscale can be pared down to Negative Mood/Physical Symptoms and Negative Self-Esteem, and component items assess symptoms of distress, such as sadness, guilt, self-loathing, and anomalies in sleep patterns, eating habits, and energy levels. The Functional Problems subscale consists of Ineffectiveness and Interpersonal Problems, and component items indicate inhibited social relationships such as peer and family relationships and maladjustment in school. Children respond on a 3-point Likert scale of 0 (no symptoms) to 2 (definite symptoms). Therefore, higher scores on the CDI-2 subscales reflect a higher incidence of depressive symptoms.

#### Multidimensional Anxiety Scale for Children, 2^nd^ edition (MASC-2) [33]

The MASC-2 was developed to measure symptoms of anxiety in children and adolescents aged 8 to 19 years. Its 50 items assess emotional, physical, cognitive and behavioural symptoms of anxiety and can be presented in terms of 6 scales and 4 subscales: Separation Anxiety/Phobias, Generalised Anxiety Disorder (GAD), Social Anxiety: Total (comprising Humiliation/Rejection and Performance Fears subscales), Obsessions and Compulsions, Physical Symptoms: Total (comprising Panic and Tense/Restless subscales) and Harm Avoidance. Children respond on a 4-point Likert scale of 0 (never) to 3 (Often). Therefore, higher scores on the MASC-2 scales reflect a higher likelihood of children experiencing symptoms of anxiety.

#### Social Emotional Assets and Resilience Scale (SEARS) [34]

The SEARS is a strength-based questionnaire for measuring positive socio-emotional competencies and assets, including peer relationships, empathy, and resilience in children and adolescents aged between 5 and 18 years. The SEARS asks youths to rate themselves on 35 statements about how they feel, think or act using a 4-point Likert scale ranging from "Never" to "Almost Always". Higher scores on the SEARS suggest better adjusted children.

### Statistical analyses

All statistical analyses were performed with R and SPSS Amos 26. We first tested the original factor structure of the CDI-2 with our data. Following a rather poor fit, an exploratory factor analysis was conducted with data from 100 participants. A confirmatory factor analysis was then conducted with the remaining data. In addition, data available for the MASC-2, CDI-2 and SEARS for 444 participants at 8.5 years of age was used to establish convergent and divergent validity of the CDI-2.

## Results

### Descriptive analyses

The CDI-2 dataset consisted of data from 732 children with 51.64% of them assessed at 8.5 years old, 10.52% of them assessed at 9 years of age and 37.84% of them assessed at 10 years old. There were 377 (51%) male and 355 (49%) female participants. Data collected on household income showed that majority of the participants (50.9%) came from households with a combined monthly income between $1999 to $5999 Singapore dollars, and only 1.9% of the participants came from households with combined monthly income below $1000 Singapore dollars. Approximately 7.7% of participants did not have available data on household income. Table 1 describes the breakdown of the demographics among our sample. At the request of an anonymous reviewer, additional demographic data comparisons were conducted between participants who completed the MASC-2 and SEARS from those who did not. No difference between the two subsets were found (S1 Table).

**Table 1:** Demographic variables (n = 730).

Demographic Variables	Categories	% among the participants
Age (Years)	8.5	51.64%
	9.0	10.52%
	10.5	37.84%
Gender	Male	51%
	Female	49%
Monthly household income (Singapore dollars)	0–999	1.9%
	1000–1999	11.9%
	2000–3999	28.0%
	4000–5999	22.9%
	More than 6000	27.6%
	Missing data	7.7%

Computed descriptive statistics for results from all three scales used in this study are presented in Table 2, along with correlations between them. The CDI-2 scores (*n* = 729) ranged from 0 to 56, and the mean score was *M* = 11.23, *SD* = 7.60. The MASC-2 scores (*n* = 444) have a minimum recorded value of 0 and a maximum recorded value of 127 across all its subscales. The mean total score on the MASC-2 in our sample was *M* = 65.74, *SD* = 22.53. The SEARS scale (*n* = 340) has values that range from 0 to 104. The average total score obtained by participants was *M* = 55.57, *SD* = 20.70. Across all three scales, the large standard deviations point to a substantial degree of variance in our sample.

**Table 2:** Descriptive statistics (Pearson’s r, mean and standard deviations).

Variable	CDI-2	MASC-2	SEARS
**CDI-2**	-		
**MASC-2**	.196*	-	
**SEARS**	-.399*	.03	-
Mean	11.228	65.741	55.574
Std. Deviation	7.604	22.526	20.702

Note.

* p < .05; CDI-2: Children’s Depression Inventory, (2^nd^ Ed.); MASC-2: Multidimensional Anxiety Scale for Children, (2^nd^ Ed.); SEARS: Social Emotional Assets and Resilience Scale

We verified the internal consistency of the CDI-2 with our sample using Kovac and MHS staff’s factor structure [31]. The original factor structure was reported to have internal consistency values ranging from .67 to .91 for the overall scale and all subscales [35]. The CDI-2 in our sample showed good overall reliability, Cronbach’s α = .854 [CI: .839, .869] (Table 3). However, a closer look at the reliability of the original CDI-2 subscales in our sample showed that subscale A (Emotional Problems) had a lower, albeit acceptable, Cronbach ‘s α = .737 [CI: .708, .764]. This points to some items as being read and interpreted differently by our participants in Singapore, consistent with previous research illustrating cultural differences in interpretation of the CDI [27, 29].

**Table 3:** CDI-2’s original factor structure reliability statistics.

Scale Reliability Statistics	Cronbach’s α
CDI-2 (overall)	.854 [CI: .829, .872]
CDI-2 Subscale A: Emotional Problems	.737 [CI: .708, .764]
CDI-2 Subscale B: Functional Problems	.763 [CI: .737, .788]

*Note*. Of the observations, pairwise complete cases were used. Removing any items did not significantly improve reliability.

### Confirmatory factor analysis

The CDI-2 dataset was first screened for missing values. There were less than 0.1% missing values–a single participant had not responded to all questions; this participant’s responses were omitted from the dataset such that the final dataset used for analysis consisted of 729 responses. We began by testing the fit of the original two-factor structure of the CDI with our dataset by performing a confirmatory factor analysis with Maximum Likelihood estimation. A significant Bartlett’s test of sphericity (p < .001) and a good Kaiser-Meyer-Olkin measure of sampling of .810 verified that our sample was adequate for factor analysis [36].

We examined the model fit using four commonly used practical fit indices: the goodness-of-fit index (GFI), the comparative fit index (CFI), the Tucker-Lewis index (TFI) and the root mean square error of approximation (RMSEA). Statisticians have recommended that the following range of values are used as a guide in interpreting fit index values [37] (Table 4).

**Table 4:** Interpreting practical fit indices.

Practical Fit Indices	Good fit	Acceptable fit	Poor fit
Root mean square error of approximation (RMSEA)	≤ .06	.06 to .08	>.10
Comparative fit index (CFI)	≥ .95	.90 to .94	< .90
Tucker-Lewis index (TLI)	≥ .95	.90 to .94	< .90
Goodness-of-fit index (GFI)	≥ .95	.90 to .94	< .90

Considering previous inconsistencies of the CDI factor structure in mind, a total of three models were specified in an initial confirmatory factor analysis: a single-factor model (Model 1), the original two-factor structure (Model 2) and a hierarchical two-factor structure (Model 3) which accounted for second-order factors that made up each of the two subscales in the CDI-2. The model fit indices used to examine the suitability of the model for our data are presented in Table 5 below.

**Table 5:** Model fit indices for CDI-2’s original factor structures (n = 729).

	Model	Model Comparison				Model Fit			
		*χ* ^*2*^	*df*	*χ*^*2*^ */ df*	RMSEA [95% CI]	TLI	CFI	ΔCFI	GFI
1	One-factor model	840.761	350	2.40	.044 [.040, .048]	.840	.852	-	.919
2	Two-factor model (original)	829.218	349	2.38	.043 [.040, .047]	.843	.855	.003	.920
3	Hierarchical two-factor model (original)	755.775	345	2.19	.040 [.037, .044]	.864	.876	.021	.928

Based on the guidelines by Hu and Bentler [37], the single-factor structure (*χ*^2^ (350) = 840.76, *χ*^2^ / df = 2.40, GFI = .919, CFI = .852, TLI = .840, RMSEA = .044 [90% CI: .040, .048]), the original two-factor structure (*χ*^2^ (349) = 829.218, *χ*^2^ / df = 2.38, GFI = .920, CFI = .855, TLI = .843, RMSEA = .043 [90% CI: .040, .047]), and hierarchical two-factor structure (*χ*^2^ (345) = 755.78, *χ*^2^ / df = 1.63, GFI = .928, CFI = .876, TLI = .864, RMSEA = .040 [90% CI: .037, .044]) had fit indices that ranged from poor to average. None of the three models had good fit indices for the CFI, TLI and the GFI.

A comparison of all three models showed that while the single-factor model and two-factor model did not differ considerably in their fit indices, the hierarchical two-factor model had the best fit indices for this Singaporean sample out of the three models we examined (Table 5). However, this was still not considered to be a good fit for our sample when looking at the average GFI (.928) and poor TLI (.864) and CFI (.876) numbers.

### Exploratory factor analysis

As the original factor structure of the CDI-2 was not found to have a very good fit with our Singapore sample, we decided to conduct an exploratory factor analysis (EFA) to examine whether a better factor structure for our data might exist. A random sample of 100 participants’ data was used to conduct the exploratory factor analysis (Group A), while a larger dataset from 629 participants was retained for the confirmatory factor analysis (Group B). A significant Bartlett’s test of sphericity, p < .001, and a good Kaiser-Meyer-Olkin measure of sampling of .781 again verified that our sample of 100 participants was adequate for factor analysis [36].

The EFA, using Maximum Likelihood estimation with oblique rotation was conducted as factors were assumed to be correlated [38]. The initial EFA revealed seven factors with Eigenvalues greater than 1. To establish the number of factors to extract, a parallel analysis [39] was also used based on the number of items included in the analysis and the number of participants comprising the sample. A Monte Carlo simulation with the same sample size (*n* = 100) and number of variables (28) as our dataset was subjected to multiple iterations and the Eigenvalues were recorded. The random Eigenvalues obtained from the Monte Carlo simulation and the seven eigenvalues from the exploratory factor analysis on our data set were then compared (Table 6). EFA-generated eigenvalues from our dataset greater than random eigenvalues generated from parallel analysis were subsequently retained for further analysis. In addition, Factor 2 (Eigenvalue 1.96) was not substantially greater than its randomly generated Eigenvalue counterpart (Eigenvalue 1.93). Therefore, we noted that there might be a one-factor solution in addition to the two-factor solution suggested by the parallel analysis, as was also noted when the model fit indices of a one-factor solution closely mirrored that of the two-factor solution (Table 6).

**Table 6:** Comparison between factor analysis-generated eigenvalues and randomly generated eigenvalues.

Factor	Factor analysis-generated Eigenvalues	Parallel analysis-generated Eigenvalues
1	7.08*	2.16
2	1.96*	1.93
3	1.60	1.82
4	1.58	1.71
5	1.38	1.60
6	1.36	1.52
7	1.18	1.42

Note.

* Factors retained for further analysis

An exploratory factor analysis with Maximum Likelihood extraction and oblique rotation was performed to assess the factor solution suggested by the parallel analysis, specifying a two-factor solution. The results of the parallel analysis, eigenvalues and a scree plot confirmed a two-factor solution, similar to the original CDI-2 factor structure. Both factors cumulatively accounted for 32% of the variance. However, in our sample, items that loaded onto Factor 1 and Factor 2 were different from the items that loaded onto each factor in the original CDI-2 subscales (Table 7).

**Table 7:** Item loadings for Factor 1 and 2 from exploratory factor analysis.

Items		Factors and Loadings	
KMO Measure of Sampling Adequacy = .781			
		1	2
9R	I feel like crying every day	.838	
21	I do not have any friends	.744	
1	I am sad all the time	.739	
4	Nothing is fun at all	.599	
5	My family is better off without me	.568	
11	I do not want to be with people at all	.549	
19	I feel alone all the time	.530	
25	I get into arguments with friends all the time	.502	
7R	All bad things are my fault	.481	
10R	I feel cranky all the time	.478	
22	I do very badly in subjects I used to be good in	.460	
6R	I hate myself	.391	
13	I look ugly	.391	
3	I do everything wrong	.364	
15R	I have trouble sleeping every night		.652
28	It is very hard to remember things		.549
24R	Nobody really loves me		.526
14R	I have to push myself all the time to do my schoolwork		.491
26R	I fall asleep during the day all the time		.452
2R	Nothing will ever work out for me		.450
20R	I never have fun at school		.441
17R	Most days I do not feel like eating		.413
23R	I can never be as good as other kids		.404
12R	I cannot make up my mind about things		.354
18	I worry about aches and pains all the time		.295
27	Most days I feel like I can’t stop eating		.295
8	I want to kill myself		.293
16	I am tired all the time		.233
	Eigenvalues	**7.076**	**1.930**
	Percentage of variance	**25.27%**	**6.90%**

As the original CDI-2 had two first-order and two second-order factors, we examined the items that loaded onto each of our factors presented in Table 7. We then performed two further exploratory factor analyses with Maximum Likelihood extraction and oblique rotation–one on each of the factors that emerged in the first EFA (Table 7), again specifying a two-factor solution. This analysis resulted in four second-order factors–two for each first-order factor.

We then examined the items that loaded onto each of our four second-order factors and named these: Negative emotion, Social isolation, Negative cognition and Vegetative symptoms. We then examined the items that comprised our two first-order factors and we named these Socio-Emotional Problems and Cognitive-Behavioural Problems to fully capture the gamut of items that comprised both first-order factors. The distribution of items across four distinct areas of child development, as revealed by our second-order factors, suggests that depressive symptoms can manifest in children in several ways, covering both internalising and externalising behaviours. The items that loaded onto each factor and their respective item loadings are presented in Table 8.

**Table 8:** First- and second-order factors from exploratory factor analysis and their item loadings.

Items		Factors and Loadings			
		Socio-Emotional Problems		Cognitive-Behavioural Problems	
		Negative Emotion	Social Isolation	Negative Cognition	Vegetative Symptoms
6	I hate myself	.602			
19	I feel alone all the time	.567			
13	I look ugly	.535			
1	I am sad all the time	.533			
9	I feel like crying every day	.523			
8	I want to kill myself	.488			
10	I feel cranky all the time	.441			
5	My family is better off without me	.435			
25	I get into arguments with friends all the time		.517		
4	Nothing is fun at all		.503		
24	Nobody really loves me		.484		
21	I do not have any friends		.463		
11	I do not want to be with people at all		.447		
20	I never have fun at school		.445		
17	Most days I do not feel like eating		.274		
22	I do very badly in subjects I used to be good in			.549	
28	It is very hard to remember things			.517	
3	I do everything wrong			.515	
23	I can never be as good as other kids			.512	
12	I cannot make up my mind about things			.484	
2	Nothing will ever work out for me			.471	
14	I have to push myself all the time to do my schoolwork			.431	
18	I worry about aches and pains all the time			.357	
7	All bad things are my fault				.464
16	I am tired all the time				.387
15	I have trouble sleeping every night				.376
26	I fall asleep during the day all the time				.314
27	Most days I feel like I can’t stop eating				.248
	Eigenvalues	2.767	2.392	2.463	1.234
	Percentage of variance	18.40%	15.90%	18.90%	9.50%
	Cumulative variance		**34.40%**		**28.40%**

### Confirmatory factor analysis

Results of the two EFA conducted earlier presents us with two possible models for the CDI-2 factor structure in the Singapore sample: 1. Two-factor model following the EFA item loadings (Model 4, Table 7) and 3. Hierarchical two-factor model with two first-order factors and two second-order factors (Model 5, Table 8). These two models were subjected to confirmatory factor analyses using the data from group B (*n =* 629*)* and compared to the single factor model (Model 1). Once again, all three models were evaluated using commonly used practical fit indices to assess model suitability for our data.

The results showed that our two-factor model (Model 4) (*χ*^2^ (349) = 687.42, *χ*^2^ / df = 1.97, GFI = .926, CFI = .878, and TLI = .867, RMSEA = .039 [90% CI: .035, .044]) had a better fit than the single-factor model (*χ*^2^ (350) = 777.67, *χ*^2^ / df = 2.22, GFI = .913, CFI = .845, and TLI = .833, RMSEA = .044 [90% CI: .040, .048]). Our two-factor model (Model 4) was also found to be a better fit for our data than the original CDI-2’s two-factor structure (Model 2). However, a closer examination of the model fit indices revealed that the hierarchical two-factor model (Model 5) (*χ*^2^ (345) = 636.27, *χ*^2^ / df = 1.85, GFI = .931, CFI = .895, and TLI = .885, RMSEA = .037 [90% CI: .032, .041]) had the best fit compared to Models 1 and 4.

The modification indices for the hierarchical two-factor model showed that it could be further improved by allowing errors to correlate. Based on the modification indices, we allowed error variances to correlate for items that loaded onto the same factor and if the MI par change was noted to be above 10. This approach was justified as the items within each factor are closely related to each other (although each item captures a distinct element of the factor) and resulted in a significantly improved goodness-of-fit (*χ*^2^ (342) = 590.96, *χ*^2^ / df = 1.73, GFI = .936, CFI = .910, and TLI = .900, RMSEA = .034 [90% CI: .029, .039]). Table 9 shows model fit indices for all four models, including the modified hierarchical two-factor model.

**Table 9:** Model fit indices for CDI-2’s factor structures from a Singaporean sample (n = 629).

No.	Model	Model Fit				Model Comparison			
		*χ* ^*2*^	*df*	*χ*^*2*^ */ df*	RMSEA [90% CI]	TLI	CFI	ΔCFI	GFI
1	One-factor model	777.67	350	2.22	.044 [.040, .048]	.833	.845	-	.913
4	Two-factor model	687.42	349	1.97	.039 [.035, .044]	.867	.878	.033	.926
5	Hierarchical two-factor model	636.27	345	1.85	.037 [.032, .041]	.885	.895	.017	.931
5a	Modified hierarchical two-factor model	590.96	342	1.73	.034 [.029, .039]	.900	.910	.015	.946

### Reliability and validity of emergent subscales

Finally, we assessed the reliability and validity of the emergent subscales using indicators of internal consistency, composite reliability (CR), and average variance extracted (AVE). Reliability of the subscales was examined using internal consistency measures of Cronbach’s alpha values and composite reliability values.

The first-order factors (Socio-emotional problems and Cognitive-behavioural problems) showed good internal reliability with our Singapore community sample, Cronbach’s α = .842 [CI: .801, .870] and Cronbach’s α = .802 [CI: .790, .872], respectively (Table 10). Individual item reliabilities suggested that removing item 17 ("Most days I do not feel like eating") from the subscale Socio-emotional Problems would improve Cronbach’s α to .907; however the overall scale reliability did not improve by removing this item. Both factors also demonstrated composite reliability values > .6 (Table 10), indicating good internal reliability.

**Table 10:** Reliability and validity indicators for emergent subscales for CDI-2.

Factor	No. of items	Cronbach’s α	Composite Reliability ^a^	Average Variance Extracted ^b^
Socio-emotional problems	14	.842 [CI: .801, .870]	.822	.760
Cognitive behavioural problems	14	.802 [CI: .790, .872]	.751	.805
**Criterion**	**-**	**> .7**	**> .7**	**> .5**

^a^ Composite reliability (CR) = (Σ factor loadings)^2^ / [(Σ factor loadings)^2^ + Σ (1 –(Σ factor loadings^2^))]

^b^ Average Variance Extracted (AVE) = (Σ factor loadings^2)^ / number of items

As all the model fit indices met the required levels, the proposed subscales were assumed to have construct validity [40]. Convergent validity is established when all values of AVE exceed .5, along with CR values exceeding .7 [40]. As such, with AVE values of our proposed subscales > .700, they were considered to have met the criteria for convergent validity.

## Discussion

The aim of this study was to examine the factor structure and psychometric properties of the CDI-2 with a Singapore community sample. Symptoms of depression in children are diverse and can manifest across multiple facets of affect, behaviour, and cognition. The CDI has emerged as the most popular measure of childhood depressive symptoms [21]. Thus, it is unsurprising that multiple researchers have analysed the factor structure and psychometric properties of the CDI to better understand how childhood depression is expressed. However, a meta-analysis of psychometric studies [30] revealed that empirically derived factor structures across different studies did not correspond to the original factor structure proposed by Kovacs [21] and indicated cross-cultural differences in the factor structure of the CDI. In addition, apart from reporting different numbers of factors, the studies in the meta-analysis also reported differences in the content and interpretation of each sub-factor. Despite changes made in the original CDI and the subsequent development of CDI-2, the factor structure of this instrument remains to be confirmed. Moreover, with only one prior study examining its factor structure in the Asian context [32], it was imperative that CDI-2 structure be evaluated in yet another Asian sample. In doing so it is interesting that the present study found a different factor structure from that found by Kim and co-workers.

We used both exploratory and confirmatory factor analyses. Our initial exploratory factor analysis revealed a hierarchical two-factor solution with good internal consistency for the two factors. This hierarchical two-factor structure was then tested with a confirmatory factor analysis and was indeed confirmed as having a good fit to data compared to other factor structures. Based on the factor loading patterns, two first-order factors and two second-order factors emerged. This finding is roughly consistent with both the original two- factor structure proposed by Maria Kovacs [31] and the only other study (to the best of our knowledge) that has evaluated the psychometric properties of the CDI-2 [32]. Thus, all three studies, including the present one, suggest that the CDI-2 measures two main dimensions.

Despite the overarching similarities, it must be noted that the original factor structure by Maria Kovacs suggested two first-order and two second-order factors; however, the factor structure proposed by Kim et al. [32] only had two factors. Furthermore, the item loadings presented by Kim and coworkers substantially differed from Kovac’s original item loadings. The item loadings presented in our study also vary significantly compared to the item loadings suggested by both Kim et al. [32] and Kovacs and MHS Staff [31] and are further discussed here. Considering the lack of empirical papers assessing the CDI-2, it is hard to situate our findings in the broad context of previous research. As such, we will discuss our results in relation to the findings of Kovacs and MHS Staff [31], but more directly in relation to the Kim et al. with an Asian sample [32].

With regards to our factor structure, only one of our second-order factors (negative cognition) corresponded to negative self-concept or low self-esteem; the latter two were both reported in the Kovacs and MHS Staff and Kim et al. studies [31, 32]. When considering the contents of each factor and individual item loadings in each factor, somatic symptoms (Item 18 “I worry about aches and pains all the time”) and concerns about food (Item 27 “Most days I feel like I can’t stop eating”) and appetite (Item 17 “Most days I do not feel like eating”) had lower item loadings compared to other items in these factors. This signifies that somatic symptoms and changes in appetite and eating behaviours may not be characteristic of childhood depression in an Asian population [41]. This is also supported by findings from Kim et al [32], the only other study that has assessed the psychometric properties of the CDI-2 in an Asian population. They also reported low item loadings for items 17, 18 and 27, which indicated that “guilty feeling, concern about somatic symptom and an increase in appetite may not reflect typical features of emotional or functional problem in Korean children and adolescent group”.

All items loading onto the Negative Emotion second-order factor included key words of “hate”, “alone”, “ugly”, “sad”, “crying”, “kill myself”, “cranky”, “better off without me”. These words are clearly reflective of the experience of negative emotions. The Social Isolation second-order factor included items that either reflected negative interactions with others (Item 25 “I get into arguments with friends all the time”) or a sense of loneliness (Item 21 “I do not have any friends”) and lack of enjoyment (Item 4 “Nothing is fun at all”). Similarly, the Negative Cognition second-order factor predominantly consisted of items which conveyed negative thoughts about oneself. Four of the five items that loaded onto our Vegetative Symptoms second-order factor correspond to Kovacs and MHS Staff’s [31] Negative Mood/Physical Symptoms sub-factor from the original CDI-2 factor structure. However, we interpreted these items as vegetative symptoms because they relate to eating and sleeping behaviours. Our first and second-order factors thus show that depressive symptoms manifest across four aspects of child development: emotional, social, cognitive and behavioural aspects.

The differences in the manifestation and expression of depressive symptoms identified in this study and the other two studies that have used the CDI-2 [31, 32] might be attributed to an interaction between culture and language [22, 25, 26]. Future studies could consider the suggestion of Bonicatto et al. [42] on how to tease out cultural influences from an interaction effect of culture and language by comparing the factor structure of the CDI-2 among individuals from different countries that speak the same language. We also suggest that future research discriminate between sources of variations in language and culture, perhaps by using bilingual respondents.

It is important to note that our results might have been affected by a few limitations. When interpreting the results presented here, it is necessary to bear in mind the age of our participants. We examined the CDI-2 responses of a cohort of 8.5 to 10.5 year olds while previous studies reported results from participants with a wider age range, e.g. Kim et al. [32] sampled participants aged 7 to 17 years old. Since the nature of the turbulent changes in emotional and psychological experience in general varies across different stages of adolescence, our results may not apply to all developmental stages in their entirety, but rather, are only applicable for this age group spanning late childhood to early adolescence. As the CDI-2 has been designed for use with a very wide range of ages, the factor structures that emerge across early adolescence and late adolescence might be very different. Woo and colleagues developed an Asian Adolescent Depression Scale, which demonstrated sound psychometric properties in a clinical and community sample of adolescents, and found four factors, namely negative self-evaluation, negative affect, cognitive inefficiency and lack of motivation [43]. Hence, negative socially oriented self-evaluation and cognitive inefficiency were important in Singaporean adolescents’ conceptualization of depression and are likely to be Asian culture-specific dimensions. In addition, it would not have been useful to examine gender differences within our sample given that such differences become apparent around 13–15 years of age [18, 19]. It would be an important next step to examine gender differences in factor structure of CDI-2 among older samples. Finally, this study only sampled Southeast Asians within Singapore, and therefore may not be generalisable to the broader Southeast Asian community.

Despite the limitations mentioned above, we believe that this study constitutes a valuable contribution to the understanding of the internal structure of the CDI-2, especially in terms of its cross-cultural uniqueness. The factor structure identified in the present study also suggests that depressive symptoms can manifest across all domains of a child’s development and provides us with insight into aspects of depression that eight to ten-year olds in Singapore struggle with. This allows educators and other specialists to tailor interventions to address specific facets of childhood depression, be they socio-emotional or cognitive-behavioural in nature. We also note the importance of more precise clinical phenotyping for the sake of investigations of underlying mechanisms, such as studies of neuroimaging or genotyping.

## Supporting information

S1 Table Demographic variables comparing MASC and SEARS completers and non-completers at year 8.5. (DOCX)

## References

[1] World Health Organization (WHO). Depression and Other Common Mental Disorders: Global Health Estimates. Geneva: World Health Organisation; 2017. https://apps.who.int/iris/bitstream/handle/10665/254610/WHO-MSD-MER-2017.2-eng.pdf

[2] Ogbo FA, Mathsyaraja S, Koti RK, Perz J, Page A. The burden of depressive disorders in South Asia, 1990–2016: findings from the global burden of disease study. BMC Psychiatry. 2018; 18(1):1–11. 30326863 10.1186/s12888-018-1918-1 PMC6192293

[3] Luby JL, Gaffrey MS, Tillman R, April LM, Belden AC. Trajectories of preschool disorders to full DSM depression at school age and early adolescence: continuity of preschool depression. Am J Psychiatry. 2014; 171:768–76. doi: 10.1176/appi.ajp.2014.13091198 24700355 PMC4103647

[4] Kovacs M, Lopez-Duran N. Prodromal symptoms and atypical affectivity as predictors of major depression in juveniles: Implications for prevention. J Child Psychol Psychiatry. 2010; 51:472–96. doi: 10.1111/j.1469-7610.2010.02230.x 20202041 PMC2921595

[5] Garber J, Rao U. Depression in children and adolescents. In: Lewis M, Rudolgh KD, editors. Handbook of Developmental Psychopathology. 3rd ed. New York: Springer; 2014. p. 489–520.

[6] Ang Y-S, Frontero N, Belleau E, Pizzagalli DA. Disentangling vulnerability, state and trait features of neurocognitive impairments in depression. Brain. 2020; 143(12):3865–77. 33176359 10.1093/brain/awaa314 PMC7805803

[7] Kessler RC, Avenevoli S, Costello J, Green JG, Gruber MJ, McLaughlin KA, et al. Severity of 12-month DSM-IV disorders in the national comorbidity survey replication adolescent supplement. Arch Gen Psychiatry. 2012; 69(4):381–9. doi: 10.1001/archgenpsychiatry.2011.1603 22474106 PMC3522117

[8] Cho SJ, Jeon HJ, Kim MJ, Kim JK, Kim US, Lyoo IK, et al. Prevalence and correlates of depressive symptoms among the adolescents in an urban area in Korea. J Korean Neuropsychiatr Assoc. 2001; 40(4):627–39

[9] Kaushik A, Kostaki E, Kyriakopoulos M. The stigma of mental illness in children and adolescents: A systematic review. Psychiatry Res. 2016; 243:469–94. doi: 10.1016/j.psychres.2016.04.042 27517643

[10] Reavley NJ, Cvetkovski S, Jorm AF, Lubman DI. Help-seeking for substance use, anxiety and affective disorders among young people: Results from the 2007 Australian National Survey of Mental Health and Wellbeing. Aust NZ J Psychiatry. 2010; 44:729–35. doi: 10.3109/00048671003705458 20636194

[11] Saluja G, Iachan R, Scheidt PC, Overpeck MD, Sun W, Giedd JN. Prevalence of and risk factors for depressive symptoms among young adolescents. Arch Pediatr Adolesc Med. 2004; 158:760–5. doi: 10.1001/archpedi.158.8.760 15289248

[12] De Los Reyes A, Kazdin AE. Informant discrepancies in the assessment of childhood psychopathology: A critical review, theoretical framework, and recommendations for further study. Psychol Bull. 2005; 131:483–509. doi: 10.1037/0033-2909.131.4.483 16060799

[13] Costello JE, Erkanli A, Angold A. Is there an epidemic of child or adolescent depression? J Child Psychol Psychiatry. 2006; 47(12):1263–71. doi: 10.1111/j.1469-7610.2006.01682.x 17176381

[14] Weiss B, Garber J. Developmental differences in the phenomenology of depression. Dev Psychopathol. 2003; 15:403–43. doi: 10.1017/s0954579403000221 12931835

[15] Zalsman G, Brent DA, Weersing VR. Depressive disorders in childhood and adolescence: An overview: Epidemiology, clinical manifestation and risk factors. Child Adolesc Psychiatr Clin N Am. 2006; 15:827–41. doi: 10.1016/j.chc.2006.05.002 16952763

[16] Watson R, Harvey K, McCabe C, Reynolds S. Understanding anhedonia: A qualitative study exploring loss of interest and pleasure in adolescent depression. Eur Child Adolesc Psychiatry. 2020; 29(4):489–99. doi: 10.1007/s00787-019-01364-y 31270605 PMC7103575

[17] Roelofs J, Braet C, Rood L, Timbremont B, van Vlierberghe L, Goossens L, et al. Norms and screening utility of the Dutch version of the Children’s Depression Inventory in clinical and non-clinical youths. Psychol Assess. 2010; 22:866–77. doi: 10.1037/a0020593 21133547

[18] Nolen-Hoeksema S, Girgus JS. The emergence of gender differences in depression during adolescence. Psychol Bull. 1994; 115(3):424–43. doi: 10.1037/0033-2909.115.3.424 8016286

[19] Townsend L, Musci R, Stuart E, Heley K, Beaudry MB, Schweizer B, et al. Gender differences in depression literacy and stigma after a randomized controlled evaluation of a universal depression education program. J Adolesc Health. 2019; 64(4):472–77. doi: 10.1016/j.jadohealth.2018.10.298 30612807 PMC6571527

[20] Moretti MM, Fine S, Haley G, Marriage K. Childhood and adolescent depression: Child-report versus parent-report information. J Am Acad Child Psychiatry. 1985; 24:298–302. doi: 10.1016/s0002-7138(09)61090-6 4008820

[21] Kovacs M. Children’s depression inventory: Manual. North Tonawanda, NY: Multi-Health Systems; 1992.

[22] Jelínek M, Květon P, Burešová I, Klimusová H. Measuring depression in adolescence: Evaluation of a hierarchical factor model of the Children’s Depression Inventory and measurement invariance across boys and girls. PLoS ONE. 2021; 16(4):e0249943 doi: 10.1371/journal.pone.0249943 33831100 PMC8031460

[23] Hodges KK, Siegel LJ, Mullins L, Griffin N. Factor analysis of the Children’s Depression Inventory. Psychol Rep. 1983; 53(3):759–63. doi: 10.2466/pr0.1983.53.3.759 6657832

[24] Saylor CF, Finch AJ, Spirito A, Bennett B. The children’s depression inventory: a systematic evaluation of psychometric properties. J Consult Clin Psychol. 1984; 52(6):955. doi: 10.1037//0022-006x.52.6.955 6520288

[25] Huang C, Dong N. Dimensionality of the Children’s Depression Inventory: Meta-analysis of pattern matrices. J Child Fam Stud. 2014; 23(7):1182–92.

[26] Craighead WE, Smucker MR, Craighead LW, Ilardi SS. Factor analysis of the Children’s Depression Inventory in a community sample. Psychol Assess. 1998;10(2):156.

[27] Burleson Daviss W, Birmaher B, Melhem NA, Axelson DA, Michaels SM, Brent DA. Criterion validity of the Mood and Feelings Questionnaire for depressive episodes in clinic and non‐clinic subjects. J Child Psychol Psychiatry. 2006; 47(9):927–34. doi: 10.1111/j.1469-7610.2006.01646.x 16930387

[28] Olorunju SB, Akpa OM, Afolabi RF. (2018). Modelling the factor structure of the Child Depression Inventory in a population of apparently healthy adolescents in Nigeria. PLoS ONE. 2018; 13(3):1–14. 10.1371/journal.pone.0193699 PMC5844540 29522568

[29] Gomez R, Vance A. Children’s Depression Inventory: Testing measurement invariance for the hierarchical factor model across children and adolescents in a clinic-referred sample. J Child Dev Disord. 2016; 2:1–8.

[30] Twenge JM, Nolen-Hoeksema S. Age, gender, race, socioeconomic status, and birth cohort differences on the Children’s Depression Inventory: A meta-analysis. J Abnorm Psychol. 2002; 111:578–88. doi: 10.1037//0021-843x.111.4.578 12428771

[31] Kovacs MMHS Staff. Children’s Depression Inventory 2nd Edition (CDI 2). Technical manual. Toronto, Canada: Multi-Health Systems; 2011.

[32] Kim H, Lee E, Hwang S, Hong S, Kim J. Psychometric Properties of the Children’s Depression Inventory-2 among a Community-Based Sample of Korean Children and Adolescents. Korean J Clin Psychol. 2018; 37:178–87.

[33] March JS. Multidimensional Anxiety Scale for Children–2^nd^ edition. Technical Manual. North Tonawanda, NY: Multi-Health Systems; 1997.

[34] Merrell KW. Social and emotional assets and resilience scales (SEARS). Lutz, FL: Psychological Assessment Resources; 2011.

[35] Bae Y. Test Review: Children’s Depression Inventory 2 (CDI 2). J Psychoeduc Assess. 2012; 30(3):304–8.

[36] Hutcheson GD, Sofroniou N. The multivariate social scientist: Introductory statistics using generalized linear models. 1^st^ ed. California: Sage; 1999.

[37] Hu LT, Bentler PM. Cutoff criteria for fit indexes in covariance structure analysis: Conventional criteria versus new alternatives. Structural Equation Modeling. 1999; 6(1):1–55.

[38] Tabachnick BG, Fidell LS. Using multivariate statistics. 5th ed. Allyn & Bacon/Pearson Education; 2007.

[39] Horn JL. A rationale and test for the number of factors in factor analysis. Psychometrika. 1965; 30(2):179–85. doi: 10.1007/BF02289447 14306381

[40] Awang Z. Structural Equation Modeling Using Amos Graphic. UiTM Press; 2012.

[41] Reddy G, van Dam RM. Food, culture, and identity in multicultural societies: Insights from Singapore. Appetite. 2020; 149:104633. doi: 10.1016/j.appet.2020.104633 32084519

[42] Bonicatto S, Dew AM, Soria JJ. Analysis of the psychometric properties of the Spanish version of the Beck Depression Inventory in Argentina. Psychiatry Res. 1998; 79(3):277–85. doi: 10.1016/s0165-1781(98)00047-x 9704874

[43] Woo BSC, Chang WC, Fung DSS, Koh JBK, Leong JSF, Kee CHY, et al. Development and validation of a depression scale for Asian adolescents. J Adoles 2004; 27(6):677–89. doi: 10.1016/j.adolescence.2003.12.004 15561310

