# Supplementary material for: Validating the Children’s Depression Inventory-2: Results from the Growing Up in Singapore Towards Healthy Outcomes (GUSTO) study
Source: PLoS One. 2023 May 25;18(5):e0286197. doi: 10.1371/journal.pone.0286197 (PMC10212172; doi:10.1371/journal.pone.0286197)
Supplement: S1 Table — (DOCX) [file pone.0286197.s001.docx]

**Supplemental Table S1. Demographic Variables comparing MASC and SEARS completers and non-completers at Year 8.5**

| **Demographic Variables** | **Categories** |  | **% among the participants** | | |  |
| --- | --- | --- | --- | --- | --- | --- |
|  |  | **MASC completers (N=450)** | **MASC non-completers**  **(N=280)** | **SEARS completers (N=340)** | **SEARS non-completers**  **(N=390)** | |
| Gender | Male | 52% | 49% | 51% | 51% | |
|  | Female | 48% | 51% | 49% | 49% | |
| Monthly household income (Singapore dollars) | 0 – 999 | 2.0% | 1.8% | 2.6% | 1.3% | |
|  | 1000 – 1999 | 12.7% | 10.7% | 12.1% | 11.8% | |
|  | 2000 – 3999 | 30.2% | 24.3% | 30.6% | 25.6% | |
|  | 4000 – 5999 | 21.8% | 24.6% | 18.8% | 26.4% | |
|  | More than 6000 | 22.0% | 36.4% | 22.4% | 32.1% | |
|  | Missing data | 11.3% | 2.2% | 13.5% | 2.8% | |

Note. MASC-2: Multidimensional Anxiety Scale for Children, (2^nd^ Ed.); SEARS: Social Emotional Assets and Resilience Scale. Participants were all 8.5 years old when these data were collected, hence, age was not compared. Chi-square tests revealed that gender did not differ between participants who did and did not complete the MASC [X^2^(1)=0.53, *p*>0.05] and SEARS [X^2^(1)<0.01, *p*>0.05]. Fisher’s exact tests revealed that household income did not differ between participants who did and did not complete the MASC (*p*>0.05) and SEARS (*p*>0.05).
